# Anti‐*Porphyromonas gingivalis* Antibody Levels in Patients With Stroke and Atrial Fibrillation: A Systematic Review and Meta‐Analysis

**DOI:** 10.1002/cre2.70041

**Published:** 2024-11-13

**Authors:** Alessandro Cannavo, Nastaran Babajani, Behrad Saeedian, Elina Ghondaghsaz, Sandro Rengo, Amirmohammad Khalaji, Amir Hossein Behnoush

**Affiliations:** ^1^ Department of Translational Medical Sciences University of Naples “Federico II” Naples Italy; ^2^ School of Medicine, Tehran University of Medical Sciences Tehran Iran; ^3^ Tehran Heart Center, Cardiovascular Diseases Research Institute Tehran University of Medical Sciences Tehran Iran; ^4^ University of British Columbia Vancouver British Columbia Canada; ^5^ Department of Neurosciences, Reproductive and Odontostomatological Sciences University of Naples “Federico II” Naples Italy

**Keywords:** antibody, atrial fibrillation, periodontitis, *Porphyromonas gingivalis*, stroke

## Abstract

**Objectives:**

Atrial fibrillation (AF) and stroke are two highly related conditions, with periodontitis and periodontal pathogens, such as *Porphyromonas gingivalis* (Pg), appearing to be the most prominent common risk factors. In this study, we evaluated studies assessing Pg infection via serum/plasma anti‐Pg antibodies in patients with AF and/or stroke.

**Material and Methods:**

Online databases (PubMed, Scopus, Embase, and the Web of Science) were screened for studies showing the association between anti‐Pg antibodies with stroke and/or AF. Relevant data were extracted, and a subsequent random‐effects meta‐analysis was performed to calculate the pooled odds ratio (OR) or standardized mean difference (SMD) and 95% confidence intervals (CIs) for Pg seropositivity or anti‐Pg antibody levels in stroke patients compared to controls.

**Results:**

Sixteen studies were included in the systematic review. Based on the meta‐analysis performed, there was no significant difference in Pg IgA and IgG levels between patients with stroke and controls (IgA: SMD 0.11, 95% CI −0.02 to 0.25, *p* = 0.1; IgG: SMD −0.12, 95% CI −1.24 to 0.99, *p* = 0.83). Similarly, no difference was observed between these groups in terms of Pg IgA and IgG seropositivity (IgA: OR 1.63, 95% CI 1.06–2.50, *p* = 0.026; IgG: OR 2.30, 95% CI 1.39–3.78, *p* < 0.001). Subsequently, we reviewed the results of six articles investigating serum or plasma IgG antibodies against Pg in patients with AF. Our results revealed a strict association between Pg infection and AF, with AF patients exhibiting either higher anti‐Pg antibody levels or a higher prevalence of positive serum Pg antibodies.

**Conclusions:**

Our study supports the clinical utility of Pg infection assessment in patients with periodontitis and those with AF and solicits more focused studies to corroborate its use in clinical settings to enhance overall outcomes, reduce the risk of complications like stroke, and help fine‐tune personalized therapies.

## 1 Introduction

Atrial fibrillation (AF) is the most typical form of cardiac arrhythmia, with a prevalence that advances with increasing age (Hindricks et al. 2021; Zoni‐Berisso et al. 2014). Comprehensive long‐term data predicted an epidemic of AF, with millions of people projected to be affected by this disorder in the United States and Europe in the following years (Miyasaka et al. 2006; Krijthe et al. 2013; Chugh et al. 2014). Importantly, due to the nature of AF as a risk factor for other conditions, these numbers are expected to impact those of other related disorders significantly. For instance, studies have demonstrated that AF increases the risk of stroke by 5–6 times, independent of other risk factors (Wolf, Abbott, and Kannel 1991, 1978). Globally, stroke represents the second leading cause of mortality, with around 8 million people's incidence occurring each year (Krishnamurthi, Ikeda, and Feigin 2020). However, despite 20%−30% of patients having AF diagnosed before their stroke, there is another 24% out of 70% of patients without known arrhythmias that can be newly diagnosed with AF (Sposato et al. 2022). Explaining why this occurs is not a simple matter. Indeed, how AF and stroke are intertwined and whether there is a common pathogenic mechanism are two big and still unresolved questions.

Several studies have supported the role of inflammation in these two disorders. Indeed, an alteration in pro‐inflammatory markers has been correlated to the risk and incidence of both stroke (Zhang et al. 2021) and AF (Nso et al. 2021; Issac, Dokainish, and Lakkis 2007; Mohtasham Kia et al. 2023; Azarboo et al. 2024). In light of this evidence, an association between periodontitis and the risk of AF and stroke has also been demonstrated (Lafon et al. 2014; Sen et al. 2021; Park et al. 2023; Chen et al. 2016). Importantly, periodontitis is a chronic oral inflammatory disorder and the sixth most prevalent disease worldwide, affecting around 11% of the population in its severe form (Kassebaum et al. 2017). Periodontitis is a consequence of oral dysbiosis, an imbalance in the microbial communities of the mouth where a dysfunctional array of pathogenic bacteria replaces the normal flora (Shaddox et al. 2012; Liccardo et al. 2019; Radaic and Kapila 2021). Among these, *Porphyromonas gingivalis* (Pg) is particularly significant, as it plays a key role in promoting inflammation and the destruction of gum tissue (Liccardo et al. 2019; Radaic and Kapila 2021; Liccardo et al. 2024; Del Giudice et al. 2021; Liccardo et al. 2020). When Pg and other periopathogens invade the gingival tissue, they can enter the bloodstream, releasing various endo‐ and exotoxins. This not only worsens the local infection and inflammation, leading to gingivitis, but also drives systemic inflammation (Liccardo et al. 2019; Radaic and Kapila 2021; Liccardo et al. 2024; Del Giudice et al. 2021; Liccardo et al. 2020; Rengo et al. 2024). Therefore, Pg has been associated with several inflammatory‐driven disorders, including cancer, cardiovascular and neurodegenerative diseases (Shaddox et al. 2012; Liccardo et al. 2019; Radaic and Kapila 2021; Liccardo et al. 2024; Del Giudice et al. 2021; Liccardo et al. 2020; Rengo et al. 2024). Due to the high prevalence of periodontitis and the myriad of consequent complications that patients face, Pg has been extensively investigated, and reports have indicated that high levels of antibody titers against this bacterium can be detected in the serum/plasma of patients affected by periodontitis or other related systemic disorders (Bender et al. 2017; Brun et al. 2021; Aoyama et al. 2019; Park et al. 2019; Mustapha et al. 2007). Furthermore, it has been shown that in patients with AF and stroke, anti‐Pg antibodies are associated with both these disorders (Hosomi et al. 2012; Nezu et al. 2022). However, to date, no study has reviewed and analyzed all the potential dissimilarities in anti‐Pg antibody levels, reported in different studies over the last decades, in AF and/or stroke patients and controls in the presence or absence of periodontitis. Hence, we conducted a systematic review and meta‐analysis to evaluate whether anti‐Pg antibody levels are significantly different in patients with stroke and AF compared to controls.

## 2 Methods

This systematic review and meta‐analysis was performed in adherence with the Preferred Reporting Items for Systematic Reviews and Meta‐Analyses (PRISMA) 2020 guidelines checklist (Page et al. 2021). Also, the protocol of this study was registered online in PROSPERO (CRD42023486511).

### 2.1 Systematic Search Strategy

Articles included in this study were identified via systematic searches of electronic databases, including PubMed, Embase, Web of Science, and Scopus, from inception to March 2024. We have chosen these databases as they are the best online databases due to their comprehensive coverage of peer‐reviewed content and advanced search capabilities. MeSH terms and keywords such as “Atrial Fibrillation” OR “Stroke” OR “cerebrovascular infarct” AND “Porphyromonas gingivalis” OR “periodontitis” were combined to elicit original studies on the association between Pg antibody titer and AF or stroke. These keywords were chosen using synonyms found in the MeSH database of PubMed and the Emtree database of Embase. Detailed search queries can be found in Supporting Information S2: Table 1.

#### 2.1.1 Inclusion and Exclusion Criteria

The inclusion criteria were as follows: (1) full‐text studies with an observational study design; (2) studies investigating the correlation between AF, stroke, and antibody against Pg, even if the primary outcome disease discussed in the article was not AF; (3) individuals older than 18 years of age of any ethnicity and both sexes; and (4) studies in which ECG findings confirmed AF diagnosis.

The exclusion criteria were as follows: (1) preclinical studies (in vivo in animals or in vitro in cells); (2) interventional studies, book chapters, reviews, and case reports; (3) duplicate studies; (4) studies with incomplete data; and (5) conference abstracts or preprints.

The inclusion and exclusion criteria were chosen to ensure that only high‐quality, relevant studies on the correlation between AF, stroke, and antibodies against Pg were analyzed. The criteria prioritize clinical applicability and minimize bias by selecting full‐text observational studies in adults with ECG‐confirmed AF and excluding preclinical, interventional, and incomplete studies. This approach strengthens the reliability and generalizability of the review's findings.

The PICO (population, intervention, control, and outcome) for selecting studies is defined as follows:

(P): patients with AF or stroke.

(I): measuring circulating serum Pg antibody titer in patients and control group.

(C): healthy individuals.

(O): could Pg antibody levels significantly differentiate patients with stroke or AF from healthy individuals?

### 2.2 Study Selection and Data Extraction

After importing selected articles derived from predefined search queries into literature management software, duplicated articles were eliminated. Subsequently, two independent reviewers (A.K. and A.H.B.) conducted title and abstract screening based on inclusion and exclusion criteria. Additionally, a full‐text review was undertaken to finalize the included articles.

One of the reviewers (E.G.) performed data extraction of included articles using a pre‐specified datasheet of the parameters, and then the extracted data were independently cross‐checked by another reviewer (B.S.). The extracted data include the following: (1) first author name, publication year, country of publication, and design of the study; (2) study publication; (3) number of participants in each group and their characteristics, including mean age ± SD, male percentage, duration of disease; (4) serum or plasma concentration of Pg antibody levels; and (5) main findings of articles.

### 2.3 Quality Assessment

The methodological quality of the studies was assessed using the Newcastle–Ottawa Scale (NOS). Two reviewers were assigned to score each article separately. Based on the NOS, potential biases were evaluated regarding selection, comparability, and outcome. Each of them was categorized as “very good” (score of 9–10), “good” (score of 7–8), “satisfactory” (score of 5–6), or “unsatisfactory” (score of 0–5).

### 2.4 Statistical Analysis

STATA (version 17.0; StataCorp) software was used for the meta‐analysis. A random‐effects (restricted maximum likelihood) model was used for the meta‐analyses. The random‐effects model was chosen because it is superior when there is heterogeneity among studies, as it accounts for variations in study design or populations. Standardized mean difference (SMD) and 95% confidence interval (CI) were calculated for comparison of the Pg antibody levels in stroke patients and control subjects. Moreover, for comparing seropositive antibody levels in patients with and without stroke, the odds ratio (OR) and 95% CI were calculated using a random‐effects meta‐analysis. In some studies where the level of the Pg antibody titer was reported as a median and interquartile range (IQR) or median and range, we used the methodological ways suggested by Luo et al. and Wan et al. to convert them into mean and SD (Wan et al. 2014; Luo et al. 2018). The heterogeneity of studies was calculated using Cochrane's Q and Higgins' *I* ^2^ tests (Higgins, 2003; Cochran 1954). The considered heterogeneity thresholds were as follows: ≤ 25% for low, 26–75% for moderate, and > 75% for high (Higgins, 2003).

## 3 Results

### 3.1 Study Selection and Baseline Characteristics

**Figure 1:**
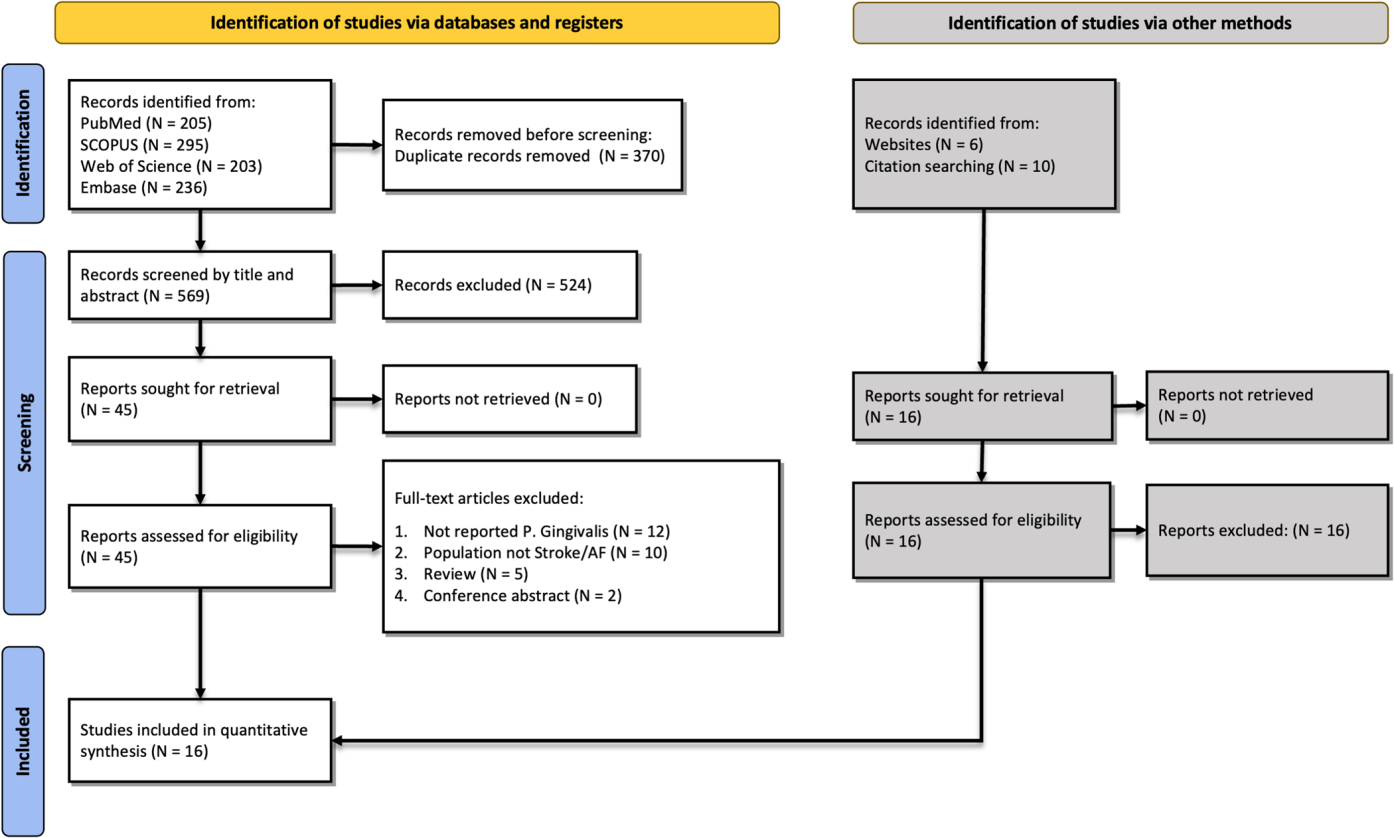
PRISMA flowchart for the search and selection process of the studies retrieved.

After database screening (PubMed, Scopus, Web of Science, and Embase), we identified 939 studies; of these, 370 studies were removed because they were duplicated (Figure 1). Other 553 studies were excluded during the title/abstract or full‐text assessment processes due to meeting the exclusion criteria we discussed previously or their inability to answer our research question in any way possible. At the end of the analysis, 16 studies were included as they fully met the criteria described in the Methods section (Hosomi et al. 2012; Nezu et al. 2022; Aoki et al. 2020; Hoshino et al. 2023; Leskelä et al. 2020; Miyauchi et al. 2021a, 2021b; Nakamori et al. 2020; Nishi et al. 2020; Palm et al. 2014, 2016; Pussinen et al. 2004, 2007; Shiga et al. 2020; Tashiro et al. 2023; Zheng et al. 2015). All of the included studies measured anti‐Pg IgA and IgG using enzyme‐linked immunosorbent assay (ELISA), except for the study by Hoshino et al. (2023), who used a chemical luminescent immunological automatic analyzer (POCube). The saliva levels of Pg were determined using quantitative real‐time PCR (qPCR) (Leskelä et al. 2020; Palm et al. 2014). Table 1 summarizes each study's characteristics and main findings. Also, as shown in Table 2, based on the NOS quality assessment system, all studies had high qualities.

**Table 1:** Characteristics of studies evaluating the Pg IgA or IgG levels in stroke or AF patients.

Author	Year	Location	Ab	Outcome	Population	Sample size	Age (mean ± SD)	Male (%)	Main findings
Aoki et al.	2020	Japan	Serum IgG	Ischemic stroke and 3‐month outcome	Patients with acute ischemic stroke	445	71.9 ± 12.3	56.0	Comparable positive IgG antibody was found between acute ischemic stroke patients with or without favorable outcomes after stroke (favorable: 11.8% vs. unfavorable: 13.3%).
Hoshino et al.	2023	Japan	Plasma IgG	AF	Cohort of patients aged 60–79 years	3091	68.6 ± 5.2	45.6	Patients with higher antibody levels had higher AF prevalence compared to lower antibody levels (3.0% vs. 1.4%; *p* = 0.005).
Hosomi et al.	2012	Japan	Serum IgG	Ischemic stroke and AF	Patients with acute ischemic stroke and healthy controls without previous stroke	209	71.1 ± 10.3	53.6	Patients with AF had significantly higher antibody compared to patients without AF (2.15 ± 0.41 vs. 1.83 ± 0.46; *p* < 0.005). However, antibody levels were comparable between stroke patients and individuals without previous stroke (stroke: 1.91 ± 0.44 logU/mL vs. no stroke: 1.86 ± 0.51 logU/mL).
Leskela et al.	2020	Germany	Serum IgG and IgA	Ischemic stroke	First‐ever ischemic stroke patients and healthy controls	198	68.6 ± 2.5	46.5	Serum IgA and IgG levels were comparable between stroke cases and healthy controls (*p* = 0.894 and 0.227, respectively).
Miyauchi et al. [JCE]	2021	Japan	Serum IgG	AF recurrence	Patients with nonparoxysmal AF/nonvalvular paroxysmal AF who underwent the first session of RFCA	596	64.9 ± 10	69.5	HigherType IV Pg serum antibody titer was associated with late recurrence (OR 1.937, 95% CI 1.301–2.884, *p* = 0.002).
Miyauchi et al. [H&V]	2021	Japan	Serum IgG	SEC	Nonvalvular AF patients who were candidates for first session of catheter ablation	569	64.9 ± 10	69.4	High‐value serum antibody titers of Pg Types II and IV were independently associated with dense SEC [Type II: adjusted OR 2.220, 95% CI 1.062–4.643, *p* = 0.02; Type IV: adjusted OR 3.169, 95% CI 1.058–6.657, *p* = 0.002].
Nakamori et al.	2020	Japan	Serum IgG	Cerebral hemorrhage growth and 3‐month outcome	Consecutive acute hemorrhagic stroke patients aged ≥ 20 years	115	71.3 ± 13.1	61.7	Comparable levels of Pg antibody were found between patients with or without hematoma growth (2.10 ± 1.03 vs. 2.01 ± 0.91, *p* = 0.75, respectively).
Nezu et al.	2022	Japan	Serum IgG	AF	Patients with acute stroke (ischemic, hemorrhagic, and TIA)	664	72.9 ± 12.6	55.1	AF patients had a higher prevalence of positive serum antibodies against Pg Type III and Pg Type V than those without AF (59.0% vs. 39.3%, *p* = 0.004 and 58.2% vs. 40.2%, *p* = 0.009, respectively).
Nishi et al.	2020	Japan	Serum IgG	Stroke and 3‐month outcome	Patients with acute stroke (ischemic or hemorrhagic)	534	71.1 ± 12.4	57.1	Detection rate for serum IgG was comparable between favorable and unfavorable outcomes groups after stroke (*p* > 0.05 for all types).
Palm et al.	2014	Germany	Serum IgG and IgA	Ischemic stroke	First‐ever ischemic stroke patients and healthy controls aged 18–80 years	198	68.7 ± 7.8	53.3	Serum IgA and IgG levels were comparable between ischemic stroke cases and healthy controls (*p* = 0.91 and 0.69, respectively).
Palm et al.	2016	Germany	Serum IgG and IgA	Ischemic stroke	First‐ever ischemic stroke patients and healthy controls	1279	66.8 ± 10.4	58.9	Detection rates for serum IgA and IgG were comparable between ischemic stroke cases and healthy controls (*p* > 0.05 for both).
Pussinen et al.	2004	Finland	Serum IgG and IgA	Stroke	Patients with fatal or nonfatal stroke and healthy controls	500	56.3 ± 5.3	45.6	Serum IgA and IgG levels were comparable between stroke cases and healthy controls in all subgroups based on history of stroke and CHD at baseline.
Pussinen et al.	2007	Finland	Serum IgG and IgA	Stroke	Population‐based cohort with or without stroke during 15‐year follow‐up	893	49.9 ± 9.5	53.9	Serum IgA and IgG levels were comparable between stroke cases and healthy controls in total population, men, and women.
Shiga et al.	2020	Japan	Serum IgG	Stroke	Patients with acute stroke (ischemic or hemorrhagic)	639	73.1 ± 12.9	55.4	Detection of Pg antibody in serum was not associated with the presence of cerebral microbleeds (*p* = 0.403). Moreover, no difference was found in frequencies of positivity for Pg in patients with mild compared to severe white matter lesions (*p* = 0.898).
Tashiro et al.	2023	Japan	Serum IgG	AF recurrence	Patients with paroxysmal AF who underwent first catheter ablation	132	62.2 ± 10.6	72.7	AF patients with periodontitis had significantly higher serum IgG levels compared to those without periodontitis (210 [71–548] vs. 94 [21–178], *p* < 0.001). Moreover, Periodontitis was independently associated with an increased risk of AF recurrence after the first catheter ablation for paroxysmal AF.
Zhang et al.	2015	China	Serum IgG	Ischemic stroke	Patients with first‐ever cerebral infarction and healthy controls	128	62 ± 12.1	78.1	A significantly higher levels of Pg IgG antibody was found in cases with cerebral infarction compared to healthy controls (11.06 ± 1.49 vs. 9.15 ± 1.70, *p* < 0.001)

Abbreviations: AF, atrial fibrillation; CHD, coronary heart disease; Pg, *Porphyromonas gingivalis*; RFCA, radiofrequency catheter ablation; SEC, spontaneous echo contrast; TIA, transient ischemic attack.

**Table 2:** Quality assessment of included studies based on the Newcastle–Ottawa Scale (NOS).

Study	Selection				Comparability	Outcome		Overall Score
	Representation	Sample size	Nonrespondents	Exposure		Outcome	Statistical test	
**Aoki et al.**	*	*	*	**	—	**	*	8
**Hoshino et al.**	*	*	*	**	—	**	*	8
**Hosomi et al.**	*	*	*	**	*	**	*	9
**Leskela et al.**	*	*	*	**	**	**	*	10
**Miyauchi et al. [JCE]**	*	*	*	**	*	**	*	9
**Miyauchi et al. [H&V]**	*	*	*	**	—	**	*	8
**Nakamori et al.**	*	*	*	**	—	**	*	8
**Nezu et al.**	*	*	*	**	**	**	*	10
**Nishi et al.**	*	*	*	**	—	**	*	8
**Palm et al.**	*	*	*	**	**	**	*	10
**Palm et al.**	*	*	*	**	**	**	*	10
**Pussinen et al.**	*	*	*	**	**	**	*	10
**Pussinen et al.**	*	*	*	**	**	**	*	10
**Shiga et al.**	*	*	*	**	—	**	*	8
**Tashiro et al.**	*	*	*	**	—	**	*	8
**Zhang et al.**	*	*	*	**	**	**	*	10

### 3.2 Meta‑Analysis of Pg Antibody Levels in Patients With Stroke vs. Controls

**Figure 2:**
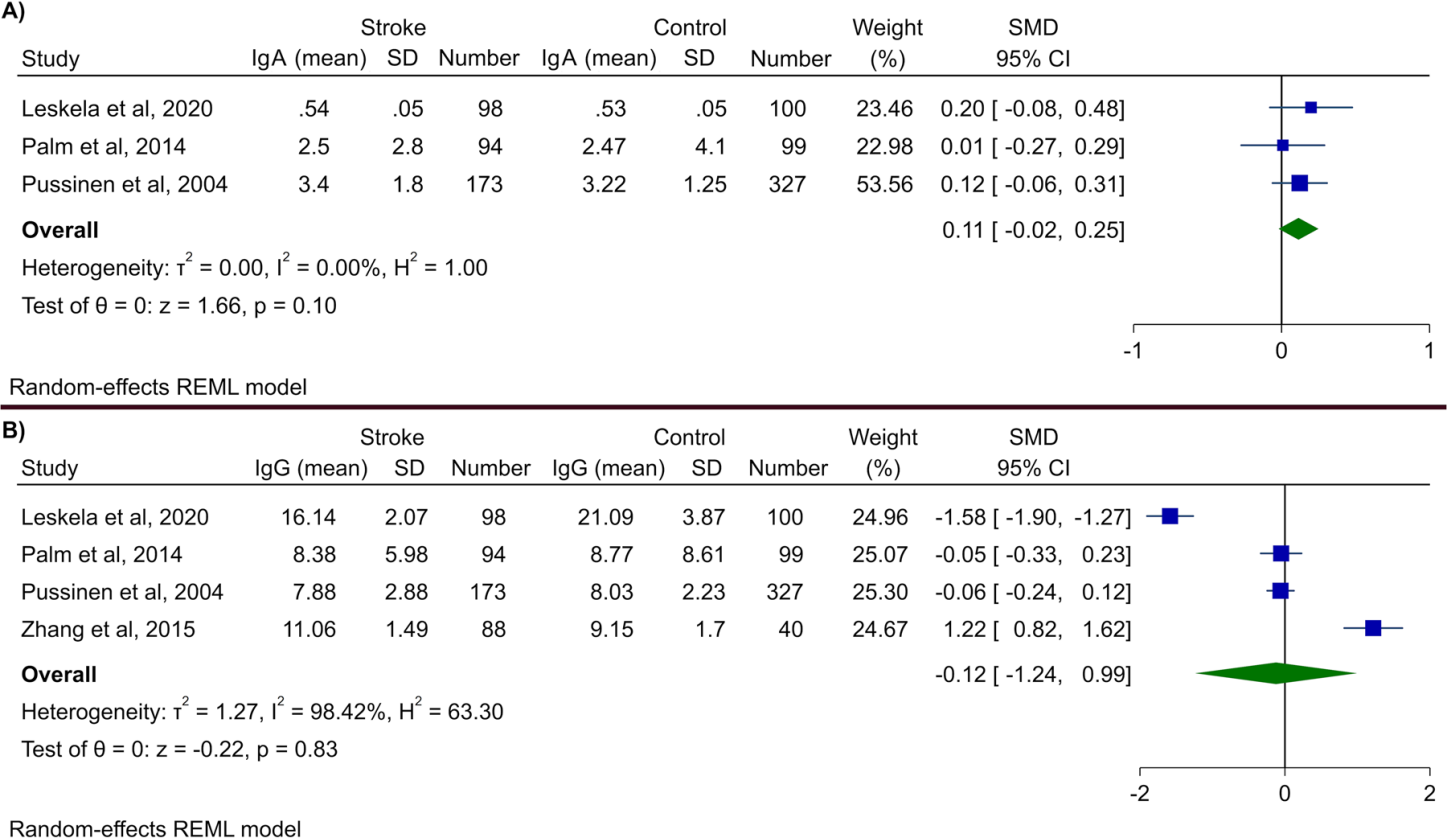
Meta‐analysis of serum levels of (A) IgA and (B) IgG in patients with stroke versus healthy controls.

We first examined the relationship between Pg IgA and IgG levels in patients with a history of stroke compared to controls. Interestingly, neither with IgA nor with IgG we found a significant association with stroke (IgA: SMD 0.11, 95% CI −0.02 to 0.25, *p* = 0.1; IgG: SMD −0.12, 95% CI −1.24 to 0.99, *p* = 0.83). There was no heterogeneity in the meta‐analysis for IgA, whereas it was high in IgG meta‐analysis, as shown by *I* ^2^ of 98%. Similarly, no significant difference was observed examining the seropositivity status of IgA and IgG in patients with stroke compared to controls (IgA: SMD 0.81, 95% CI 0.39 to 1.65, *p*‐value = 0.56; IgG: SMD 1.13, 95% CI 0.94–1.36, *p* = 0.19). The heterogeneities were 93% and 0% for IgA and IgG seropositivity analyses, representing high and no heterogeneity, respectively. Figure 2A,B shows the Forest plots for these analyses.

### 3.3 Pg Antibodies in Stroke Patients

**Figure 3:**
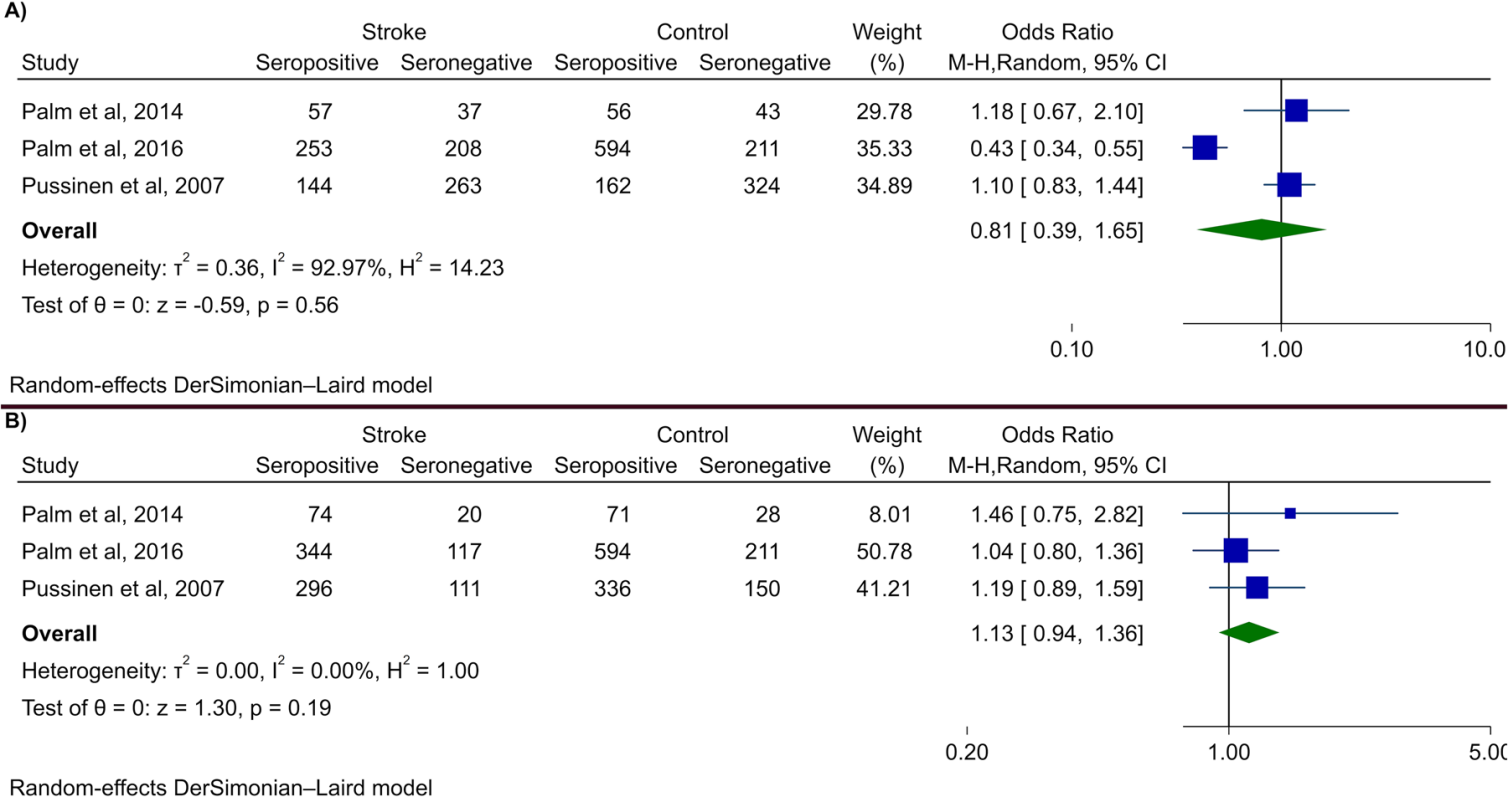
Meta‐analysis of seropositivity of (A) IgA and (B) IgG in patients with stroke versus healthy controls.

Multiple studies revealed that there were no significant differences in serum IgG (Hosomi et al. 2012; Leskelä et al. 2020; Palm et al. 2014; Pussinen et al. 2004) or IgA titers (Leskelä et al. 2020; Palm et al. 2014; Pussinen et al. 2004), as well as seropositivity (Palm et al. 2014, 2016; Pussinen et al. 2007), among patients with or without stroke. However, the study by Zhang et al. (2015) observed significantly higher IgG levels in acute cerebral infarction patients than healthy controls. In addition, these authors reported that Pg IgG titer correlated with the intima‐media thickness of the common carotid arteries (IMT‐CCA), which is an essential indicator of carotid atherosclerosis (left: *r* = 0.306, *p* = 0.004; right: *r* = 0.241, *p* = 0.024). Three studies (Aoki et al. 2020; Nakamori et al. 2020; Nishi et al. 2020) examined reported that serum IgG titers were comparable regardless of favorable or unfavorable outcomes during 3‐month follow‐ups, encompassing patients with ischemic (Aoki et al. 2020) or hemorrhagic stroke (Nakamori et al. 2020) or those diagnosed with either condition (Nishi et al. 2020). For their part, Pussinen et al. (2007) reported that men with a history of cardiovascular disease (CVD) had higher Pg IgA and IgG levels than CVD‐free controls (IgA: *p* = 0.03; IgG: *p* = 0.108). Interestingly, this study also reported a sex difference in the prediction ability of Ig between males and females of incident stroke. Indeed, although IgA seropositivity predicted a higher chance of developing stroke in males, high IgG levels represented a significant risk factor for stroke in females (IgA: OR 1.63, 95% CI 1.06–2.50, *p* = 0.026; IgG: OR 2.30, 95% CI 1.39–3.78, *p* = < 0.001). Figure 3A,B illustrates the forest plot for meta‐analysis of IgA and IgG seropositivity in patients with stroke and controls.

### 3.4 Pg Antibodies in AF Patients

Several studies demonstrated a significant association between serum (Hosomi et al. 2012; Nezu et al. 2022) and plasma (Hoshino et al. 2023) IgG levels and a higher prevalence of AF. Miyauchi et al. (2021b) showed that Pg Type IV serum IgG antibody titer significantly correlated with late recurrence of AF within 12 months (OR 1.937, 95% CI [1.301–2.884], *p* = 0.002), whereas other types (I, II, III, and V) showed no association. Next, the authors observed that those patients presenting a high value of Pg Type IV (mean + 3 SD or greater) presented with a higher recurrence rate of AF incidence of previous cerebral infarction, CHADS2 score, and oxygen desaturation index compared to those with low values (Miyauchi et al. 2021b). In another study, Nezu et al. (2022) analyzed a cohort of 234 acute stroke patients with (117) or without (117) AF. Interestingly, these authors reported that among IgG antibodies recognizing the Type I–V fimbriae subunit (FimA) of Pg, only those against the Types III and V resulted in AF prevalence in stroke patients (Nezu et al. 2022). However, no significant differences were observed between patients with sustained AF and those with paroxysmal AF in serum titers of Pg IgG FimA Types III and V. In addition, these authors examining positivity for this species according to the duration of AF (less than 1 year and over 1 year) reported that patients with AF for more than 1 year had a higher frequency of positivity for Pg (FimA Type III) than those with AF for less than 1 year (71.1% vs. 50.0%, *p* = 0.047).

Next, Hoshino et al. (2023), in a population of 3091 participants aged 60–79 years, observed that those with higher antibody levels against Pg had more than two‐fold higher odds of having AF (OR 2.13, 95% Cl 1.38–5.14, *p* < 0.005). In addition, these authors reported that higher plasma IgG levels were associated with lower median age, lower obesity prevalence, and higher prevalence of history of heart failure. However, no significant association was found with hypertension, dyslipidemia, diabetes mellitus, history of myocardial infarction, sex, and excessive drinking (Hoshino et al. 2023). In contrast, Hosomi et al. (2012) in their study reported that serum anti‐Pg antibodies were higher in male patients with drinking habits and AF compared to those without these conditions.

## 4 Discussion

AF and stroke are two strictly associated conditions. Although we have gained valuable information on how these disorders generate and progress in the last several decades, there have been fewer advances in our understanding of the molecular mechanisms responsible for their association.

Currently, several meta‐analyses and systematic reviews have demonstrated a higher incidence of AF (Leelaviwat et al. 2023), cerebral ischemia, and stroke in subjects with periodontitis (Lafon et al. 2014; Leira et al. 2017; Sfyroeras et al. 2012; Janket et al. 2003). Indeed, periodontal pathogens are causative of oral and systemic inflammation, and periodontitis acts within the same chronic inflammatory model seen in AF and stroke (Liccardo et al. 2019). In addition, among the pathogenic mechanisms proposed linking periodontitis to AF and stroke, it appears that oral pathogen‐induced atherosclerosis is one of the most prominent. For instance, here we have examined the study by Zheng et al. (2015), who reported a correlation between the intima‐media thickness of the common carotid arteries, an essential indicator of carotid atherosclerosis, which can partly explain the correlation between Pg and stroke, observed by the authors. For this reason, periodontal pathogens, particularly Pg, have been differently assessed to test their correlation with AF and stroke. Among the tools used to detect the presence of this bacterium, serum/plasma antibodies titer against Pg has been investigated in different studies, and in this systematic review and meta‐analysis, we pooled all the data available in the literature.

Six articles (Hosomi et al. 2012; Wan et al. 2014; Hoshino et al. 2023; Miyauchi et al. 2021a, 2021b; Tashiro et al. 2023) examined serum or plasma Pg IgG titer in patients with AF, and all concluded that patients with AF exhibited higher anti‐Pg antibody levels or a higher prevalence of positive serum antibodies. Interestingly, studies examining the recurrence of AF and periodontitis suggested an independent association with an increased risk of AF recurrence after the first catheter ablation for paroxysmal AF (Miyauchi et al. 2021b). However, although these conclusions corroborate those obtained by a systematic review published in 2022 by Leelapatana and Limpuangthip (2022), supporting poor oral health associated with new‐onset or recurrent AF, we could not perform a meta‐analysis due to differences in subjects (prevalence or recurrence) and antibody sampling methods.

Conversely, we were able to analyze the correlation between seropositivity or level of antibody IgG and IgA with a stroke; collectively, our analysis did not provide any significant results. For instance, the studies examining Pg IgA levels revealed that these were comparable between stroke cases and healthy controls (SMD 0.11, 95% CI [−0.02; 0.25], *p* = 0.1). Similar results were observed when comparing the studies examining Pg IgG levels between patients with stroke and controls, with no difference observed between these groups (SMD –0.12, 95% CI [−1.24; 0.99], *p* = 0.83). In addition, one of the studies included came to the same conclusions, showing similar levels of anti‐Pg antibody between hemorrhagic stroke patients with or without hematoma growth (2.10 ± 1.03 vs. 2.01 ± 0.91, *p* = 0.75, respectively).

However, despite the considerable relevance of the present study's findings, we must not overlook the potential association between Pg infection and stroke for several reasons. Indeed, it is worth noting that stroke represents the major complication of AF (Elsheikh et al. 2024). Therefore, more focused studies should be performed to analyze the incidence of stroke in AF patients in correlation with Pg infection. In addition, previous studies like the one by Ghizoni et al. (2012) demonstrated the association between Pg infection and stroke, but these studies were not included in our analysis as they used alternative methods (e.g., PCR and RT‐PCR). Finally, two studies included in our analysis demonstrated contrasting results supporting a solid correlation between Pg infection and stroke. For instance, Pussinen et al. (2004) provided serological evidence that Pg infection, seen as elevated IgA‐antibody levels, predicted a recurrent stroke in individuals with a history of stroke or coronary heart disease at baseline. However, Zheng et al. (2015) reported significantly higher levels of Pg IgG antibody in cases with cerebral infarction compared to healthy controls (11.06 ± 1.49 vs. 9.15 ± 1.70, *p* < 0.001). These discrepancies demand more investigations that are necessary to confirm or refute the association between stroke and Pg infection. These should be assessed using multiple methods, such as antibody titer, DNA, and mRNA, to detect the presence of Pg.

Although this study provided valuable insights, six main limitations should be considered when interpreting the results of our research. First, not all included articles had healthy control groups, restricting their use in the meta‐analysis. Second, the meta‐analysis findings may have limited generalizability to broader populations, as the included studies were limited in scope. Third, our study includes many observational studies, which present inherent biases in observational data. These include selection bias due to the lack of randomization, the presence of confounding factors that may not be adequately controlled, and information bias due to inaccuracies in measuring exposure or outcomes, which can affect results. Fourth, the high heterogeneity observed in the IgG meta‐analysis (*I*² of 98%) suggests substantial variability across the studies, which could impact the robustness of the pooled results. This raises essential questions about the consistency of methodologies, populations studied, and other potential confounding factors that may have contributed to the outcomes. In particular, differences in the patient population should be included among the potential confounders. Indeed, the demographic characteristics of the included studies may vary significantly in terms of factors such as ethnicity, age, sex, comorbidities, and disease severity. These variations may profoundly affect each patient's immune responses and antibody production, leading to different outcomes observed.

Another vital aspect is the distinct roles in immune responses that IgG and IgA play. Indeed, IgG is more prevalent in the bloodstream, whereas IgA is primarily found in mucosal areas, including the oral cavity (~12.5 mg/mL IgG and 2.2 mg/mL IgA) (Plomp et al. 2018). Therefore, the presence of Pg in periodontal tissues and systemically can stimulate a more pronounced IgG production as part of a systemic inflammatory response, whereas IgA levels may not rise to the same extent due to its localized (salivary) action. In this context, a recent study by Svärd et al. (2023) analyzing a cohort of patients with rheumatoid arthritis (a periodontitis‐related systemic disorder) demonstrated that production of IgA against Pg gingipains in the salivary glands was not accompanied by systemic antibody production supporting the localized mucosal activity of this antibody. Moreover, IgG production may be indicative of prolonged or systemic Pg exposure, which shifts the immune response toward a more generalized defense (Kulshrestha, Srinivasa, and Biswas 2013). In contrast, IgA may dominate in the early or localized phases of infection, where mucosal barriers are more active (Kulshrestha, Srinivasa, and Biswas 2013). Thus, the differential immune responses observed in our study may reflect the distinct roles of these antibody classes in addressing localized versus systemic infection. However, if Pg infection is persistent and diffused systemically, the immune system may shift from producing IgA to generating a more “systemic” IgG response. This aspect should be evaluated in a more in‐depth basic study that can clarify the different responses of IgA and IgG in stroke and AF.

Fifth, the methodological variability in measuring anti‐Pg antibodies across the studies may differ in assay sensitivity, specificity, and sample types (serum vs. plasma) as well as timing of sample collection that may impact antibody levels, as the immune response can vary over time. These methodological differences can introduce variability in the results, potentially masking genuine associations. Similarly, other infections, systemic inflammation, or medications that could affect immune responses may be another confounding factor that needs to be consistently controlled through studies. Finally, we acknowledge that the limited number of studies analyzed presents a critical limitation affecting our findings' overall analysis and interpretation.

Taken together, these limitations not only emphasize the need for future studies aiming at comparing antibody levels but also lead to the possibility of bias in the analyses. For instance, we were not able to assess publication bias in the analyses due to the low number of analyzed studies and the fact that funnel plots and Begg's and Egger's tests are not sensitive in analyses with less than 10 studies. Hence, the chance of publication bias remains, and this should be focused on in future research.

## 5 Conclusion

This study is the first to assess anti‐Pg antibody levels as a marker of Pg infection and periodontitis in patients with AF and/or stroke through systematic review and meta‐analysis. Among the pathogens examined, Pg is undoubtedly the most important and tightly related to AF (Liccardo et al. 2019). In contrast, our analysis did not show any significant association with stroke. Pg is currently recognized as the primary dominant gram‐negative bacterium associated with the development of periodontitis and is the critical pathogenic bacteria studied in oral microbiology (Ge et al. 2022).

Undoubtedly, future studies are warranted to comprehend better the complex relationship between Pg infection, periodontitis, and the risk of AF and stroke. However, this study underscores the importance of increasing awareness among scientists and clinicians about the need for a broader understanding of how periodontitis and periodontal pathogen infection can impact whole systemic health. Accomplishing this will provide more preventive measures for systemic disorders, including AF, and CVD in general, but will lower the economic burden of these conditions on the health system. For this reason, effective health policies should incorporate oral hygiene and periodontal disease screening into AF management strategies to improve overall outcomes and reduce the risk of complications like stroke.

## Author Contributions

A.C., B.S., and N.B. conceptualized, wrote the original draft, and reviewed and edited the document. E.G. and S.R. wrote the original draft, performed data curation, and reviewed and edited the document. A.K. and A.H.B. visualized, supervised, performed a formal analysis, and reviewed and edited the document. All authors read and approved the final manuscript.

## Ethics Statement

The authors have nothing to report.

### Consent

The authors have nothing to report.

## Conflicts of Interest

The authors declare no conflicts of interest.

## Supporting information

Supporting information.

Supporting information.

## Data Availability

The authors have nothing to report.
