# Supplementary material for: Anti‐Porphyromonas gingivalis Antibody Levels in Patients With Stroke and Atrial Fibrillation: A Systematic Review and Meta‐Analysis
Source: Clin Exp Dent Res. 2024 Nov 13;10(6):e70041. doi: 10.1002/cre2.70041 (PMC11558155; doi:10.1002/cre2.70041)
Supplement: Supplementary file 2 — Supporting information. [file CRE2-10-e70041-s001.docx]

**Supplementary Materials**

***Supplementary Table 1.*** *Search strategy for each database*

| **Query** | | **Results**  **(March 5, 2024)** |
| --- | --- | --- |
| ***PubMed*** | | |
| #1 | (“Atrial Fibrillation”[tw] OR “Atrial Fibrillations”[tw] OR “Auricular Fibrillation”[tw] OR "Persistent Atrial Fibrillation"[tw] OR "Persistent Atrial Fibrillations"[tw] OR "Familial Atrial Fibrillation"[tw] OR "Familial Atrial Fibrillations"[tw] OR "Paroxysmal Atrial Fibrillation"[tw] OR "Paroxysmal Atrial Fibrillations"[tw] OR “AF”[tw] OR “AFib”[tw] OR “Atrial Fibrillation”[MeSH]) | 129,300 |
| #2 | (“Stroke”[tw] OR “cerebrovascular infarct*”[tw] OR “cerebrovascular event*”[tw] OR “CVA”[tw] OR “transient ischemic attack*”[tw] OR “TIA”[tw] OR “brain ischemia”[tw] OR “brain infarc*”[tw] OR “brain hypoxia”[tw] OR "Cerebrovascular Disorders"[Mesh] OR "Brain Ischemia"[Mesh] OR "Hypoxia-Ischemia, Brain"[Mesh] OR "Ischemic Attack, Transient"[Mesh] OR "Stroke"[Mesh] OR "Stroke, Lacunar"[Mesh] OR "Infarction, Posterior Cerebral Artery"[Mesh] OR "Brain Stem Infarctions"[Mesh] OR "Infarction, Middle Cerebral Artery"[Mesh] OR "Infarction, Anterior Cerebral Artery"[Mesh]) | 645,800 |
| #3 | (“bacteroides gingivalis”[tw] OR “porphyromonas”[tw] OR “gingivalis”[tw] OR “P. gingivalis”[tw] OR “periodontal bacter*”[tw] OR “periodontal pathogen*”[tw] OR “periodontal microb*”[tw] OR “periodontal infection*”[tw] OR “oral bact*”[tw] OR “oral microb*”[tw] OR “oral infecti*”[tw] OR “oral pathogen*”[tw] OR (“periodont*”[tw] AND “antibody”[tw]) OR (“periodont*”[tw] AND “IgG”[tw]) OR “Porphyromonas gingivalis"[MeSH]) | 29,792 |
| #4 | #1 OR #2 | 741,483 |
| **#5** | **#3 AND #4** | **205** |
| ***Scopus*** | | |
| #1 | (TITLE-ABS-KEY(“Atrial Fibrillation”) OR TITLE-ABS-KEY(“Atrial Fibrillations”) OR TITLE-ABS-KEY(“Auricular Fibrillation”) OR TITLE-ABS-KEY("Persistent Atrial Fibrillation") OR TITLE-ABS-KEY("Persistent Atrial Fibrillations") OR TITLE-ABS-KEY("Familial Atrial Fibrillation") OR TITLE-ABS-KEY("Familial Atrial Fibrillations") OR TITLE-ABS-KEY("Paroxysmal Atrial Fibrillation") OR TITLE-ABS-KEY("Paroxysmal Atrial Fibrillations") OR TITLE-ABS-KEY(“AF”) OR TITLE-ABS-KEY(“AFib”)) | 222,572 |
| #2 | (TITLE-ABS-KEY(“Stroke”) OR TITLE-ABS-KEY(“cerebrovascular infarct*”) OR TITLE-ABS-KEY(“cerebrovascular event*”) OR TITLE-ABS-KEY(“CVA”) OR TITLE-ABS-KEY(“transient ischemic attack*”) OR TITLE-ABS-KEY(“TIA”) OR TITLE-ABS-KEY(“brain ischemia”) OR TITLE-ABS-KEY(“brain infarc*”) OR TITLE-ABS-KEY(“brain hypoxia”) OR TITLE-ABS-KEY(“cerebral infarc*”)) | 683,838 |
| #3 | (TITLE-ABS-KEY(“bacteroides gingivalis”) OR TITLE-ABS-KEY(“porphyromonas”) OR TITLE-ABS-KEY(“gingivalis”) OR TITLE-ABS-KEY(“P. gingivalis”) OR TITLE-ABS-KEY(“periodontal bacter*”) OR TITLE-ABS-KEY(“periodontal pathogen*”) OR TITLE-ABS-KEY(“periodontal microb*”) OR TITLE-ABS-KEY(“periodontal infection*”) OR TITLE-ABS-KEY(“oral bact*”) OR TITLE-ABS-KEY(“oral microb*”) OR TITLE-ABS-KEY(“oral infecti*”) OR TITLE-ABS-KEY(“oral pathogen*”) OR (TITLE-ABS-KEY(“periodont*”) AND TITLE-ABS-KEY(“antibody”)) OR (TITLE-ABS-KEY(“periodont*”) AND TITLE-ABS-KEY(“IgG”))) | 36,024 |
| #4 | #1 OR #2 | 859,308 |
| **#5** | **#3 AND #4** | **295** |
| ***Embase*** | | |
| #1 | (“Atrial Fibrillation”:ti,ab,kw OR “Atrial Fibrillations”:ti,ab,kw OR “Auricular Fibrillation”:ti,ab,kw OR "Persistent Atrial Fibrillation":ti,ab,kw OR "Persistent Atrial Fibrillations":ti,ab,kw OR "Familial Atrial Fibrillation":ti,ab,kw OR "Familial Atrial Fibrillations":ti,ab,kw OR "Paroxysmal Atrial Fibrillation":ti,ab,kw OR "Paroxysmal Atrial Fibrillations":ti,ab,kw OR “AF”:ti,ab,kw OR “AFib”:ti,ab,kw) | 206,462 |
| #2 | (“Stroke”:ti,ab,kw OR “cerebrovascular infarct*”:ti,ab,kw OR “cerebrovascular event*”:ti,ab,kw OR “CVA”:ti,ab,kw OR “transient ischemic attack*”:ti,ab,kw OR “TIA”:ti,ab,kw OR “brain ischemia”:ti,ab,kw OR “brain infarc*”:ti,ab,kw OR “brain hypoxia”:ti,ab,kw) | 550,947 |
| #3 | (“bacteroides gingivalis”:ti,ab,kw OR “porphyromonas”:ti,ab,kw OR “gingivalis”:ti,ab,kw OR “P. gingivalis”:ti,ab,kw OR “periodontal bacter*”:ti,ab,kw OR “periodontal pathogen*”:ti,ab,kw OR “periodontal microb*”:ti,ab,kw OR “periodontal infection*”:ti,ab,kw OR “oral bact*”:ti,ab,kw OR “oral microb*”:ti,ab,kw OR “oral infecti*”:ti,ab,kw OR “oral pathogen*”:ti,ab,kw OR (“periodont*”:ti,ab,kw AND “antibody”:ti,ab,kw) OR (“periodont*”:ti,ab,kw AND “IgG”:ti,ab,kw)) | 30,396 |
| #4 | #1 OR #2 | 706,307 |
| **#5** | **#3 AND #4** | **236** |
| ***Web Of Science*** | | |
| #1 | (TS=(“Atrial Fibrillation”) OR TS=(“Atrial Fibrillations”) OR TS=(“Auricular Fibrillation”) OR TS=("Persistent Atrial Fibrillation") OR TS=("Persistent Atrial Fibrillations") OR TS=("Familial Atrial Fibrillation") OR TS=("Familial Atrial Fibrillations") OR TS=("Paroxysmal Atrial Fibrillation") OR TS=("Paroxysmal Atrial Fibrillations") OR TS=(“AF”) OR TS=(“AFib”)) | 160,108 |
| #2 | (TS=(“Stroke”) OR TS=(“cerebrovascular infarct*”) OR TS=(“cerebrovascular event*”) OR TS=(“CVA”) OR TS=(“transient ischemic attack*”) OR TS=(“TIA”) OR TS=(“brain ischemia”) OR TS=(“brain infarc*”) OR TS=(“brain hypoxia”) OR TS=(“cerebral infarc*”)) | 457,026 |
| #3 | (TS=(“bacteroides gingivalis”) OR TS=(“porphyromonas”) OR TS=(“gingivalis”) OR TS=(“P. gingivalis”) OR TS=(“periodontal bacter*”) OR TS=(“periodontal pathogen*”) OR TS=(“periodontal microb*”) OR TS=(“periodontal infection*”) OR TS=(“oral bact*”) OR TS=(“oral microb*”) OR TS=(“oral infecti*”) OR TS=(“oral pathogen*”) OR (TS=(“periodont*”) AND TS=(“antibody”)) OR (TS=(“periodont*”) AND TS=(“IgG”))) | 31,599 |
| #4 | #1 OR #2 | 581,964 |
| **#5** | **#3 AND #4** | **203** |
| **Total records** | | **939** |
| **Total records after removing duplicates** | | **569** |
